# Investigating Oral Cancer Awareness Among Medical and Dental Students: A Cross-Sectional Study in the South Canara Region of India

**DOI:** 10.1155/tswj/8814749

**Published:** 2024-12-28

**Authors:** Shubham Agarwal, Nidhi Manaktala, Srikant Natarajan, Karen Boaz

**Affiliations:** Department of Oral Pathology and Microbiology, Manipal College of Dental Sciences Mangalore, Manipal Academy of Higher Education, Manipal, Karnataka, India

**Keywords:** cancer awareness, dental students, medical students, oral cancer

## Abstract

Oral cancer is responsible for increased mortality, especially in the Indian subcontinent. Habits like smoking and tobacco chewing are among the most common causes of oral cancer. Previously, these habits were seen mainly in the elderly; however, the trends have seemed to increase in the younger generation. Awareness regarding oral cancer is essential during the early years of a healthcare expert for effective diagnosis. This study aimed to investigate oral cancer awareness among medical and dental students. A cross-sectional study including 144 medical and 112 dental undergraduate students was designed. A validated 12-question questionnaire on oral cancer awareness was distributed to medical and dental undergraduate students. The collated data were analyzed statistically using tests of proportion. Dental students were more likely to examine oral mucosa routinely (*χ*^2^ = 9.585, *p*=0.002) and observe oral lesions like proliferative growth (*χ*^2^ = 71.763, *p* < 0.001), while medical students reported higher incidences of poor oral hygiene (*χ*^2^ = 7.667, *p*=0.006) and tobacco usage (*χ*^2^ = 6.337, *p*=0.012). Both groups expressed a need for more education on oral cancer, with dental students showing a stronger preference (*χ*^2^ = 7.526, *p*=0.006). Referral to an oral surgeon was preferred among both groups. The dental students felt a lack of sufficient knowledge on oral cancer. It was observed that the knowledge and information regarding cancer-related symptoms was more among medical students. However, overall awareness regarding oral cancer was higher in dental students compared to medical students. It was concluded that medical students exhibited more knowledge about oral cancer causes, while dental students were more aware of its signs and symptoms. Educational interventions should be introduced early to reduce diagnostic delays and prevent cancer progression.

## 1. Introduction

Oral cancer (OC) ranks as the 16th most prevalent malignancy impacting the lips and oral cavity, encompassing the oropharynx, hypopharynx, and larynx [1]. It ranks as the 15th most prevalent cause of mortality worldwide, with significant heterogeneity based on age, gender, race, ethnicity, and socioeconomic status [2]. Despite the existence of several treatment options, the overall five-year survival rate for OC remains approximately 50% [3]. OC, however rare in the Western world, is prevalent in high-risk nations [4]. In 2017, South Asia recorded the highest incidence, death, and national burden of OC [5]. OC ranks third among all malignancies in India and accounts for about 40% of total cancer-related mortality. Approximately 70,000 new cases and more than 48,000 deaths connected with OC occur each year. OC is quite prevalent in India, ranking as the second most common malignancy among males and the fourth among women. The prevalence escalates with age and is more prevalent in men [6, 7].

Oral squamous cell carcinoma (OSCC) was identified as the sixth most prevalent variety in 2016, accounting for 90% of head and neck malignancies [8, 9]. While OSCC can originate from anatomical sites in the head and neck, it predominantly manifests in the oral cavity [10]. The etiology of cancer is mostly linked to detrimental behaviors, including betel nut consumption, smokeless tobacco use, and alcohol abuse, as well as factors such as hereditary predisposition and excessive sun exposure [11]. The prevalence of OC among the younger demographic has lately increased due to filthy sexual practices associated with specific DNA viruses, including human papillomavirus and herpes simplex virus type I [8, 12]. Furthermore, due to the absence of a compulsion for cancer registration in India, the actual incidence and mortality rates are likely to be underestimated [13].

Recognizing the predisposing factors is crucial for facilitating early diagnosis and preventing cancer progression and subsequent mortality. OC can be discovered early by methods such as screening high-risk populations, opportunistic screening in primary healthcare settings, and the prompt identification and reporting of signs and symptoms by individuals and healthcare professionals to confirm a diagnosis [14]. This strategy is highly beneficial, but a lack of information and social stigma may hinder patients from seeking care at an early stage. Limited awareness correlates with reduced survival rates in patients with OCs [7]. Consequently, heightened knowledge of the disease and improved access to healthcare services will facilitate early diagnosis, mitigate disease development, and enhance prevention efforts. Encouraging regular dental exams is recommended to enable the opportunistic evaluation of potential indications and symptoms. Furthermore, this approach would need dentists and other primary healthcare workers to be well trained to recognize the early signs of OC. This study aimed to assess the knowledge and attitudes of dental and medical students regarding OC and to enhance their understanding of the associated hazards.

## 2. Materials and Methods

A cross-sectional study was initiated on obtaining approval from the Institutional Ethics Committee at Manipal College of Dental Sciences, MAHE, Mangalore, bearing reference number 16117, from January 2022 to June 2022. A convenience sampling method was used to enroll the undergraduate students of II-year medical (*n* = 144, Kasturba Medical College, Mangalore) and dental (*n* = 112, Manipal College of Dental Sciences, Mangalore) fraternities. The rationale for selection of II-year participants was to evaluate their awareness prior to the exposure to theoretical and clinical knowledge and evaluation of cancer and related features. The participants were thoroughly instructed about the study objectives before handing out the printed questionnaire. Permissions to administer the questionnaire were obtained from the Heads of the respective institutions. Before administering the printed questionnaire to individual participants, written consent was documented from each participant to ensure their voluntary and active participation in the study. The participants were well-informed that their personal information would be held strictly confidential.

The questionnaire employed was adapted from a published study by Carter and Ogden, to which permission was sought from the authors before use [15]. The questionnaire was revalidated for content, scope, and appropriateness by three experts before being utilized in our study. It was agreed to use the questionnaire in its original format as prescribed by Carter and Ogden. The questionnaire collected confidential personal information such as name, gender, and institutional affiliation details. The questionnaire had 12 questions, with ten close-ended questions and two open-ended questions, in English. The data obtained were compiled and analyzed using SPSS (IBM SPSS 20.0, Chicago, USA) software. Gender and streams being categorical variables, the association of the students' awareness of gender and streams was correlated with the components of the questionnaire using the chi-square test. The data for each question were depicted using tests of proportion. The global significance was set at *p* < 0.05.

## 3. Results

Two hundred fifty-six participants were enrolled (144 medical and 112 dental) for the study. All participants had effectively filled out the questionnaire and returned the same within the allotted timeframe. In the medical sample, there were about 84 females and 60 males, while the dental sample had around 66 females and 46 males. All the participants involved in the study were aged between 18 and 21 years. The questionnaire data were analyzed and are reported in Table 1.

### 3.1. Evaluation of Oral Mucosa

The routine evaluation of the oral mucosa was significantly more frequent among medical graduates, with 50.40% reporting this practice compared to only 30.40% of dental graduates (*p*=0.002). Some questions showed borderline significance, such as whether they screen the oral mucosa if patients are in high-risk categories (*χ*^2^ = 3.107, *p*=0.078), or the presence of ulcers (*χ*^2^ = 3.22, *p*=0.073); these results suggest trends that may warrant further investigation. Fewer dental students (44.40%) than medical students (58.80%) reported screening high-risk patients when they do not routinely examine the oral mucosa, and dental students related ulcer to be more related to malignancy than the medical students.

### 3.2. Awareness and Enquiry of Habits

There was no difference in awareness regarding tobacco chewing between the two groups. However, smoking habits were significantly more often included in history-taking by dental graduates (69.9%) than medical graduates (50%). Similarly, dental graduates were more likely to enquire about poor oral hygiene (23.3%) compared to medical graduates (8.2%) (*p*=0.006).

### 3.3. Counseling on Risk Factors

Dental graduates were significantly more likely to advise patients on the risk factors of OC, with 99.1% doing so compared to 88.3% of medical graduates (*p*=0.001).

### 3.4. Exposure to Oral Lesions

Ironically, medical graduates had significantly higher exposure to examining patients with oral lesions (33.6%) compared to dental graduates (9.6%) (*p* < 0.001). This could be attributed to the fact that clinical postings start early on for the medical students as compared to dental students.

### 3.5. Awareness of Clinical Features of OC

Both groups felt adequately informed about the clinical appearance of OC. However, dental graduates demonstrated greater awareness of clinical features such as white patches, proliferative growth, cauliflower-like growths, and pedunculated growths, compared to medical graduates. Medical graduates predominantly identified ulcers as a clinical feature but were less aware of other signs of OC.

### 3.6. Referral Patterns

Dental graduates were more likely to refer patients with suspected OC to oral and maxillofacial surgeons (55.7%) compared to medical graduates (39.8%). Medical graduates, however, were equally likely to refer patients to oral medicine departments (27.6%) as dental graduates (27.4%).

### 3.7. Need for Additional Training

A significantly higher proportion of dental graduates (94.1%) expressed the need for additional teaching on OC compared to medical graduates (82%). Both groups preferred seminars and informational packets as their preferred modes of learning seminars over lectures. Overall gender-related differences were not significant in the present study, and both males and females exhibited equal understanding awareness and understanding related to all the abovementioned parameters.

## 4. Discussion

This study aims to elucidate the behaviors and attitudes of dental and medical students regarding OC, a lethal oral disease, and to enhance knowledge of its related risk factors at an early age. Our study found that medical graduates examined the oral mucosa substantially more than their dentistry and medical student counterparts. This could be attributed to the fact that clinical exposure for medical students start from the II year itself. Approximately 49.6% of medical graduates and 69.6% of dentistry graduates do not assess the oral mucosa. Dental graduates must enhance their thoroughness in examining the oral mucosa and teeth. Early recognition of the significance of teeth and their related structures will enhance their comprehension of deviations from normalcy.

Our study noted that, within high-risk categories, oral cavity screening rose to 58.8% in medical groups and 44.4% in dental groups, compared to 50.4% and 30.4% for routine examinations conducted by both groups, respectively. The data indicate that medical students engage in a clinical program starting in their second year, but dental students are introduced to a clinical environment only in their third year. This may explain the results for parameters 1 and 2, wherein medical students might have assessed patients' oral cavities, a scenario that was improbable for dentistry undergraduates. Our findings are consistent with previously published literature [16, 17]. Dental students should be encouraged to periodically visit a clinical setting to enhance their understanding of normal and pathological conditions of the oral cavity. Furthermore, this exposure can be expanded for spontaneous screening of high-risk patients during routine oral examinations, even at the community level.

When comparing information about the etiology of OC, both medical and dental students demonstrated sufficient understanding of risk factors such as tobacco use and smoking. Medical graduates predominantly screened for chewing tobacco as the most prevalent high-risk category, whereas dental graduates focused more on smoking. Poor oral hygiene prompted more dentists to assess the oral mucosa than medical graduates. Nonetheless, the students had a limited understanding of additional risk factors related to alcohol usage and viral infections [15]. Dental students had a greater enthusiasm for informing their patients about the risk factors associated with OC than their medical counterparts. This is likely due to the fact that a dental graduate's area of specialization is the oral cavity, in contrast to that of medical students. A study indicates that medical students exhibit a deficient attitude toward OC, attributed to little exposure during their educational experience [18]. To foster awareness, broader comprehension must be imparted to the undergraduate students. The majority of dental students had negative responses regarding the opportunity to evaluate patients with oral lesions, in contrast to medical students. This disparity may be attributed to the variations in curricula among the fraternities.

The dentistry group had much greater awareness of the clinical signs and symptoms of OC compared to the medical students. Nonetheless, both medical and dental graduates perceived themselves as sufficiently versed about their clinical presentation. Both student groups concurred that the ulcerative lesions are symptomatic of cancer. Nevertheless, dental graduates exhibited superior awareness of the malignancy of white patches (74.7%), proliferative growth (74.7%), verrucous development (35.6%), and pedunculated growth (27.6%). Nonetheless, literature reports exhibit inconsistencies contingent upon geographical location and several study characteristics [18]. This may indicate enhanced awareness among dental students. The importance of early clinical indicators should be underscored to facilitate prompt diagnosis.

In our study, medical graduates predominantly recommend referring patients to a physician when oral lesions are identified, in contrast to dentistry graduates. Enhancing understanding of the dentist's function in OC detection and prevention within the medical curriculum may elevate knowledge of streamlined referral mechanisms. Our findings indicated that, upon graduation, medical and dental students would preferentially refer patients to specialists in oral and maxillofacial surgery or oral medicine. Nonetheless, 12.2% of the medical graduates will refer patients to ENT specialists. Medical graduates possess greater awareness of ENT, a subject that is undervalued in dentistry, potentially explaining the unfamiliarity observed among dental students. Both groups concurred that the optimal recommendation for an OC patient is to an oral and maxillofacial surgeon. The outcome concurred with the extant literature [15]. A knowledgeable and proficient healthcare team is essential to reduce the disparities between patient screening and referral to a specialist. This knowledge will facilitate an efficient referral system to avert unnecessary diagnostic and preventive delays. Medical and dentistry students similarly lack adequate understanding concerning the prevention and detection of mouth cancer and require additional information on the subject. The dentistry graduates would desire substantially more information about OC compared to the medical graduates. The information pack was the predominant request among medical graduates in contrast to seminars requested by dental graduates. Numerous findings have been documented by numerous authors within the pertinent literature.

Soares et al. concluded that students had a good knowledge of the etiology of OC and were alert in their examinations to the possibility of detecting malignant lesions. However, they noted that the clinical features of the lesion were not sufficiently clear to the students. They suggested that knowledge of OC, in particular its clinical presentation, needed to be reinforced throughout the undergraduate dental course to enable raising suspicions and making an early diagnosis of lesions [19]. Anushya, Dhanraj, and Keerthi also opined the necessity for an improvement of the teaching program regarding oral examination [20]. Rai et al. and Chan et al. reported general lack of awareness about OC and its associated risk factors among student cohorts (dental and medical) of the Singaporean population and Malaysian population, respectively [21, 22]. They too emphasized upon the need for targeted education and to provide training to improve diagnostic skills for every dental undergraduate. Similar sentiments were echoed in studies conducted by Fotedar et al. (2015), Kumar and Ak, Keser and Pekiner, and Saleem, Mahmoud and Joseph. Given considerable morbidity and effect on quality of life, initiatives to prevent the condition and enhance early detection are essential [23–26]. Nazar, Ariga, and Shyama studied knowledge, attitudes, and practices regarding OC among recently graduated dentists in Kuwait and found that majority of dentists were knowledgeable and aware of the numerous aspects of OC. They also concluded that the training programs in OC education must be prioritized and reinforced, with a particular emphasis on early detection and prevention. They strongly advised that dentists participate in continuing education programs and seminars to increase their understanding of the risk factors and diagnosis of OC [27].

The findings of the present study indicated a disparity in knowledge and awareness of OC between the two study groups. The current curricular design does not allow dental students to see patients during their second year of training, in contrast to medical students, who begin patient interactions at the start of their second year. Engaging second-year dental students as observers in dental screening camps may address this gap. Medeiros et al. assessed knowledge about OC among dental students and Primary Health Care Dentists in Brazil and concluded the need for proper training to examine and identify signs of OC [28]. Algudaibi et al. studied the difference of knowledge of OC between dental and medical practitioners and found that dental practitioners were found to be more knowledgeable about the high OC risk sites and predisposing factors than medical practitioners [29]. As the adage goes “nipping the evil in the bud,” understanding risk factors along with diagnostic instruments to identify early symptoms can help alleviate the burden of OC. There were certain limitations in the study. A convenience sampling was performed; hence, the entire student community of both the institutes was not subjected to the questionnaire. A larger community was not provided with the questionnaire due to the higher risk of dropouts. Only the Mangalore campus of Manipal Academy of Higher Education was enrolled in the study for strategic access to the geographical location.

## 5. Conclusion

Within the study limitations, it was concluded that the medical students had more knowledge about OC in terms of causes. In contrast, the dental students had more awareness of the signs and symptoms related to OC. It was also seen that dental students sought more information related to OC than medical students. Through this study, an attempt to elicit knowledge and awareness was made, and an introduction of an important step was attempted. The information gained through this questionnaire helps the students change perspectives regarding OC. An educational intervention or early exposure to dental/oral health screening camps should be implemented promptly to sensitize the students and familiarize them with the initial signs and symptoms of OC. The study's extended scope involves conducting further analyses involving multiple institutions and larger sample size to investigate factors affecting OC awareness among medical and dental students, including demographic variables, curriculum content, and clinical exposure, to gain a deeper understanding of the underlying reasons for the observed disparities in knowledge and practices between these groups.

## Figures and Tables

**Table 1:** Results of the Oral Cancer Awareness Questionnaire.

Questions	Answer choices	N	Groups				Chi square	*p* value
			Medical		Dental			
			Count	Column (%)	Count	Column (%)		
1. Do you examine patient's oral mucosa routinely	No	139	68	49.60	71	69.60	9.585	0.002
	Yes	100	69	50.40	31	30.40		

2. If your answer is no to question 1, do you screen the oral mucosa if the patients are in high-risk categories?	No	73	33	41.20	40	55.60	3.107	0.078
	Yes	79	47	58.80	32	44.40		

3a. Tobacco chewing	Absent	30	11	11.20	19	26.00	6.337	0.012
	Present	141	87	88.80	54	74.00		

3b. Smoking	Absent	71	49	50.00	22	30.10	6.798	0.009
	Present	100	49	50.00	51	69.90		

3c. Bad hygiene	Absent	146	90	91.80	56	76.70	7.667	0.006
	Present	25	8	8.20	17	23.30		

4. When you have graduated will you advise patients about the risk factors for oral cancer	No	17	16	11.70	1	0.90	10.586	0.001
	Yes	226	121	88.30	105	99.10		

5. Have you had the opportunity to examine patients with oral lesions?	No	185	91	66.40	94	90.40	19.03	< 0.001
	Yes	56	46	33.60	10	9.60		

6. As regards the clinical appearance of oral cancer do you feel?	Very well informed	17	8	7.00	9	9.20	1.519	0.678
	Well informed	26	12	10.40	14	14.30		
	Adequately informed	121	66	57.40	55	56.10		
	Poorly informed	49	29	25.20	20	20.40		

7a. Ulcer	Absent	61	35	43.20	26	29.90	3.22	0.073
	Present	107	46	56.80	61	70.10		

7b. White patches	Absent	62	38	46.90	24	27.60	6.729	0.009
	Present	106	43	53.10	63	72.40		

7c. Proliferative growth	Absent	95	73	90.10	22	25.30	71.763	< 0.001
	Present	73	8	9.90	65	74.70		

7d. Cauliflower-like growth	Absent	130	74	91.40	56	64.40	17.458	< 0.001
	Present	38	7	8.60	31	35.60		

7e. Pedunculated growth	Absent	136	73	90.10	63	72.40	8.532	0.003
	Present	32	8	9.90	24	27.60		

7f. Others	Absent	168	81	100.00	87	100.00	.	.
	Present	0	0	0.00	0	0.00		

8. On finding oral lesions the patient should preferably visit	Dentist	139	46	34.80	93	90.30	73.601	< 0.001
	Physician	96	86	65.20	10	9.70		

9. When you have graduated where would you refer a patient if you suspected an oral malignancy?	Plastic surgery	7	7	5.70	0	0.00	23.934	0.001
	ENT	17	15	12.20	2	1.90		
	Oral and maxillofacial surgery	108	49	39.80	59	55.70		
	Oral medicine	63	34	27.60	29	27.40		
	Dentist	25	10	8.10	15	14.20		
	General practitioner	4	4	3.30	0	0.00		
	Other	5	4	3.30	1	0.90		

10. Do you feel that you have sufficient knowledge concerning prevention and detection of oral cancer?	No	173	95	73.60	78	77.20	0.39	0.532
	Yes	57	34	26.40	23	22.80		

11. Would you like more information or teaching on oral cancer?	No	29	23	18.00	6	5.90	7.526	0.006
	Yes	201	105	82.00	96	94.10		

12. If so which format would you prefer? Circle one or more	Information pack	91	52	49.50	39	40.60	2.373	0.305
	Lectures	37	20	19.00	17	17.70		
	Seminars	73	33	31.40	40	41.70		

## Data Availability

The datasets generated and/or analyzed during the current study are not publicly available due to institutional policy but are available from the corresponding author upon reasonable request.
